# Neighborhood conditions in a Swedish context-Two studies of reliability and validity of virtual systematic social observation using Google Street View

**DOI:** 10.3389/fpsyg.2023.1020742

**Published:** 2023-01-27

**Authors:** Ingela Clausén Gull, Sabina Kapetanovic, Åsa Norman, Laura Ferrer-Wreder, Tina M. Olsson, Lilianne Eninger

**Affiliations:** ^1^Department of Psychology, Stockholm University, Stockholm, Sweden; ^2^Department of Social and Behavioral Studies, University West, Trollhättan, Sweden; ^3^Department of Clinical Neurosciences, Karolinska Institute, Stockholm, Sweden; ^4^Department of Social Work, University of Gothenburg, Gothenburg, Sweden; ^5^School of Health and Welfare, Jönköping University, Jönköping, Sweden

**Keywords:** contextual resources, neighborhoods, developmental assets, child and youth development, observational method, systematic social observation, Google street view

## Abstract

**Introduction:**

The goal of these studies was to investigate the reliability and validity of virtual systematic social observation (virtual SSO) using Google Street View in a Swedish neighborhood context.

**Methods:**

This was accomplished in two studies. Study 1 focused on interrater reliability and construct validity, comparing ratings conducted in-person to those done using Google Street View, across 24 study sites within four postal code areas. Study 2 focused on criterion validity of virtual SSO in terms of neighborhoods with low versus high income levels, including 133 study sites within 22 postal code areas in a large Swedish city. In both studies, assessment of the neighborhood context was conducted at each study site, using a protocol adapted to a Swedish context.

**Results:**

Scales for Physical Decay, Neighborhood Dangerousness, and Physical Disorder were found to be reliable, with adequate interrater reliability, high consistency across methods, and high internal consistency. In Study 2, significantly higher levels of observed Physical Decay, Neighborhood Dangerousness, and signs of garbage or litter were observed in postal codes areas (site data was aggregated to postal code level) with lower as compared to higher income levels.

**Discussion:**

We concluded that the scales within the virtual SSO with Google Street View protocol that were developed in this series of studies represents a reliable and valid measure of several key neighborhood contextual features. Implications for understanding the complex person-context interactions central to many theories of positive development among youth were discussed in relation to the study findings.

## 1. Introduction

Contextual resources within neighborhoods play an important role in child and youth development (e.g., Fauth et al., 2007; Leventhal and Brooks-Gunn, 2011; Browning et al., 2013; Riina et al., 2013; McCoy et al., 2015; Gard et al., 2021). Neighborhood contexts and their characteristics provide developmental assets critical both in terms of connecting youth to others in the community as well as providing a setting in which young people can attain personal goals and thrive (Tolan et al., 2016). Assessments of contextual resources and neighborhood characteristics, such as safety, orderliness, and the condition of buildings, is often carried out through in-person Systematic Social Observation (SSO) or though surveys where key neighborhood features are reported by members of a community (e.g., Odgers et al., 2015; Bader et al., 2017; Hoeben et al., 2018). As a complement to these types of assessments of physical neighborhood characteristics, researchers are developing tools to measure the features of neighborhoods that are efficient and feasible. For example, SSO can be performed virtually with support from geographical tools like Google Street View (GSV; Odgers et al., 2012). As contextual resources are of particular interest in research concerning child and youth development, we examined, in two studies, the psychometric properties of a virtual SSO method with GSV to determine the reliability and validity of the method for assessment of physical neighborhood characteristics that are relevant in a Swedish neighborhood context.

### 1.1. Wider relevant theory

From a positive youth development approach, communities can break the isolation of families and individuals through connecting them to other key contextual assets, provide a place and opportunity to work towards wider goals (e.g., greater equity and access to opportunities) that benefit community members through collective efforts (Benson et al., 2012). Communities can also provide young people with an understanding of what it means to be a contributing member of their community through socialization experiences (Benson et al., 2012). In the developmental assets framework, external assets that speak directly to the type and character of resources to be found within communities are within the asset domains of external support, empowerment, as well as boundaries and expectations (Benson et al., 2012). Illustrations of external, community relevant, assets involve young people’s reports of the actual relational support they receive from adults (not including their parents), other relational resources in their immediate environment (e.g., availability of adult role models, engagement of neighbors in setting boundaries on youth public behavior) as well as the actual roles that young people have taken on within their community (e.g., community service/social action opportunities for young people to engage in).

The external assets that are most relevant to the present study regard safety. In the assets framework (e.g., Benson et al., 2012), youth report on their perception of safety in the key developmental contexts of home, school, and neighborhood. While the young person’s perception of neighborhood safety is of clear value, this perspective can be complemented by other indicators of neighborhood safety (as it is done in the present study) as well as documentation of other possible resources within the everyday context of children and youth. Indeed, a present-day shortcoming of the positive youth development approach, in general, is a lack of attention to observational assessments of assets/resources in the context of development as a needed step to fill in the picture of the person context interactions that are operating at the heart of positive forms of adaptation and behavior over time.

Staying within developmental science, but now drawing from meta-theoretical perspectives of person context interactions, one can also look to ecological systems perspectives (Bronfenbrenner, 1979) to unpack how the neighborhood context is operating in order to have both direct and indirect importance to individual development of children and youth across domains of development (e.g., Leventhal and Brooks-Gunn, 2011; Gard et al., 2021). Accordingly, neighborhoods offer a proximal setting in which children build their skills and competencies (McCoy et al., 2015).

In addition to the aforementioned perspectives from developmental science positive youth development, the present study also considered the wider, interdisciplinary study of neighborhoods and how aspects of neighborhoods relate to behavior and well-being. For example, the importance of the neighborhood as context has also been described from several different problem-oriented perspectives within the fields of antisocial behavior and community crime and violence exposure (e.g., Browning et al., 2013).

### 1.2. Importance of studying neighborhoods

Neighborhood disorder and unsafety can pose a threat for physical and mental health at the individual and community levels (Browning et al., 2013). Several studies have indicated that in neighborhoods with a high concentration of poverty, including lack of safety, poor housing and increased neighborhood disorder, children and youth can be subjected to higher level of psychosocial challenges, such as internalizing and externalizing problems (Jones et al., 2011) including for example anti-social behavior (Odgers et al., 2015), detachment from parents (Singh and Ghandour, 2012), as well as have negative associations with resident children’s cognitive skills, such as reading (Vinopal and Morrissey, 2020). Indeed, children living in neighborhoods with perceived safety concerns (e.g., presence of garbage or litter in streets/sidewalks, poor/dilapidated housing, and vandalism/graffiti) have about two times higher odds of serious behavioral problems than children living in more favorable neighborhoods, even after adjustment for household poverty status (Singh and Ghandour, 2012).

Thus, the wider research literature in this area speaks broadly to the importance of the quality of the physical environment to behavior and well-being. Moreover, the physical environment may not only have importance to the development of academic and social competence in children and youth but may also have a long-term bearing on brain development and development of cognitive skills (Evans, 2006; Mcewen and Mcewen, 2017; Gard et al., 2021). On the other hand, children residing in more advantaged neighborhoods seem to benefit in terms of their socioemotional skills, verbal ability, and school achievement (Leventhal and Brooks-Gunn, 2000; Leventhal et al., 2003). Such inequalities in the neighborhood contexts are particularly evident in regard to institutional resources, such as childcare and schools as these institutions are often resourced and operated generally shaped by the structure of the neighborhood (Jencks and Mayer, 1990). As children spend a great amount of time in learning environments, where they interact with peers and other adults, studying neighborhoods in which preschools and schools are situated would provide important insight into children’s contexts of development and their possibilities to thrive.

### 1.3. Systematic social observation as a method to describe neighborhoods

Investigating contextual resources, including physical characteristics in a neighborhood, can be difficult. Often, resident surveys have been used to assess the general quality and social processes of neighborhoods (Sampson et al., 1997) as well as physical characteristics in a neighborhood (for an overview see Brunton-Smith, 2018). These methods are useful in that they provide important information on residents’ subjective perspectives of the neighborhoods they live in. Yet, resident surveys can provide insight into characteristics of a neighborhood and contextual resources, but also has disadvantages in that there is a potential risk for non-response, selective non-representative responses, and for socially desirable answers (Mooney et al., 2017; Hoeben et al., 2018). The contextual resources within neighborhoods can also be described in terms of indicators of socioeconomic status, derived from government administrative data. However, more nuanced neighborhood characteristics, like disorder, may not be well reflected by census or other registry based data, and administrative boundaries may misrepresent variability across neighborhoods (Mooney et al., 2014; Brunton-Smith, 2018). The contributions as well as limitations of resident surveys and census data described here, point to the need for a diversity of measurement approaches and a wide range of constructs needed to understand the totality of neighborhood contexts, which lends support for efforts to develop several methods including those that rely on observations of a neighborhood (Hoeben et al., 2018). In this area, different observational methods have been developed to provide an observed ecological assessment of neighborhood characteristics across a diversity of different constructs (e.g., Raudenbush and Sampson, 1999; Sampson and Raudenbush, 1999).

Systematic Social Observation (SSO), is a direct observational method in which raters walk a block or street segment within a neighborhood to perform an assessment of contextual conditions, such as housing conditions and evidence of social and physical disorder (Sampson and Raudenbush, 1999; Brunton-Smith, 2018). The systematic aspect of SSO is that the rating features have theoretical and practical relevance and observers are trained to reliably observe these key features. SSO seems to be especially suitable for the assessment of physical disorder, given that signs of physical disorder are relatively stable over time compared to social disorder, in that social disorder reflects events that can occur randomly or sporadically in time (Sampson and Raudenbush, 1999). In addition, studies using SSO, especially within the fields of criminology and sociology, have over time demonstrated that neighborhood physical features and social disorder, independently coded by multiple raters, also correlate with resident perceptions of physical disorder and fear of crime (Brunton-Smith, 2018). Thus, SSO can be recognized one of a diversity of methods to obtain estimates of ecological neighborhood characteristics (Brunton-Smith, 2018).

SSO can be conducted live and in person or *via* virtual methods. In-person SSO can be both time consuming and costly, as raters have to travel to the study areas to collect data (Odgers et al., 2012). With support of recent digital geographical tools such as Google Street View (GSV), SSO can also be performed virtually and with fewer resources than in-person observations (Odgers et al., 2012). SSO using GSV, where raters take a virtual walk through neighborhoods, is an unobtrusive and easily accessible way to collect neighborhood characteristics within natural settings (Odgers et al., 2012; Marco et al., 2017). The use of GSV also allows for the use of the same source data across different sites and geographical locations, as the data collection approach for GSV images are consistent (Brunton-Smith, 2018). Although the date (time period or time stamp) for available GSV images can vary, from months up to years, street level images are also provided for many populated regions within Western countries.^1^

SSO has advantages but also has limitations such as observer bias, both within and between observers, particularly SSO that is carried out in person (Hoeben et al., 2018). However, to perform SSO virtually may reduce the risk for intra-observer bias, i.e., socialization and fatigue, as raters do not spend a lot of time travelling and walking around in the neighborhood in person, and rather can conduct the observations individually, with less effort and during a shorter time period. Furthermore, the use of virtual SSO reduces the risk for bias due to reactivity, i.e., that the mere presence of observers may affect resident’s behavior of disorder. The biggest risk for observation bias, both with in person and virtual SSO, is differences in observations between raters (inter-observer bias), due to differences in observers’ personal characteristics and prior experiences (Hoeben et al., 2018). Inter-observer bias can be minimized with careful design of the instrument and appropriate training as well as clear instructions for raters about what features to observe and how to record them (Brunton-Smith, 2018).

Recently developed instruments for virtual SSO are considered to be reliable and cost-effective methods to study neighborhood conditions, as suggested by studies from the United States (Clarke et al., 2010; Bader et al., 2017) and the United Kingdom (Odgers et al., 2009, 2012; Griew et al., 2013). For example, studies have compared virtual and in-person assessment of street-level neighborhood characteristics, such as physical disorder (Odgers et al., 2012; Mooney et al., 2017), land use and recreational facilities (Clarke et al., 2010), physical decay and neighborhood dangerousness (Odgers et al., 2012), and results indicated that virtual assessment was reliable and could be used as a substitute for or supplement to in-person observations. A recommendation is however that study areas should be small and observed features relatively stable over time in order to achieve high reliability (Less et al., 2015). Moreover, Odgers et al. (2012) reported high levels of observed agreement between four independent raters about the presence of disorder, physical decay, and assessments of neighborhood safety, which also corresponded with local resident reports and demonstrated an association with census-defined indices of socioeconomic status. Considering that virtual SSO is a context dependent method, and as research to date has mostly focused on urban areas in United States and United Kingdom, the generalizability of this method to other social and cultural contexts has yet to be fully examined (Odgers et al., 2012; Griew et al., 2013).

### 1.4. Swedish neighborhoods

**Figure 1:**
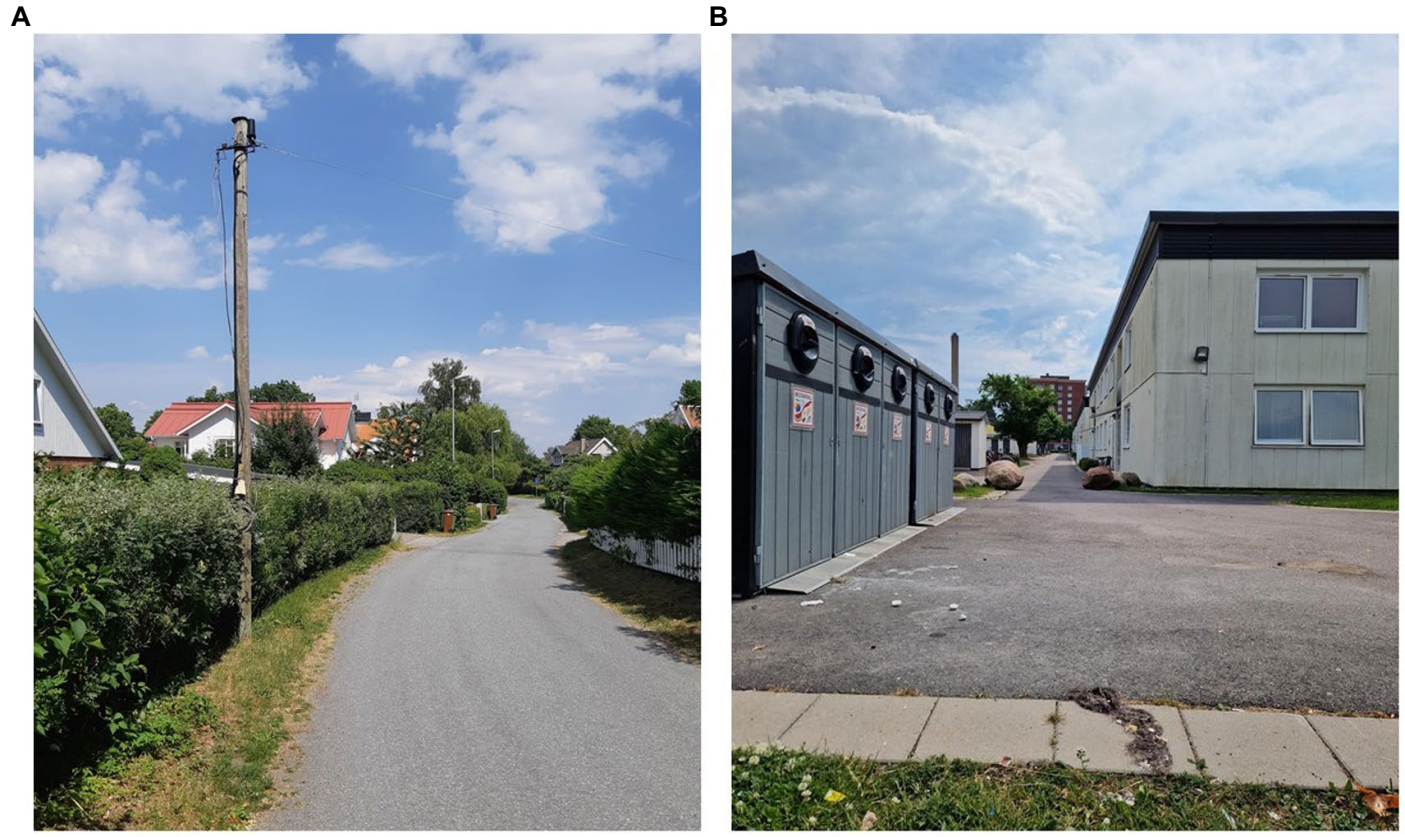
Examples of Swedish neighborhoods (not part of the present study), similar to what can be seen in GSV. **(A)** Single-family houses. Photo: Ingela Clausén Gull. **(B)** Multi-family housing. Photo: Sabina Kapetanovic.

Swedish neighborhoods are embedded within a wider social and cultural context. That is, Sweden is a social welfare state, with a social and political fabric that supports the rights of children and families to thrive under the best living conditions that are practically possible. In Sweden, neighborhoods often include areas with single-family houses and areas with multi-family housing, including tenant-owned cooperatives and first and second-hand rental apartments, see Figure 1. In general, streets and sidewalks in these areas are well kept, and less variability in these aspects could therefore be expected. Moreover, neighborhoods are typically diverse in character and land use, and include much vegetation and wooded areas, also in close vicinity to residential units. Therefore, Swedish neighborhoods examined in this study can be considered to be a mix of urban and suburban built-up areas. In some of the neighborhoods, particularly those with multi-family housing and rental apartments, there can be signs of social and economic segregation, including poorer economic resources, overcrowding and high concentration of residents with immigrant backgrounds. Such segregation can also be noticeable in schools. In order to meet children’s educational needs, some municipalities reallocate resources to schools with low-performing and socially and economically disadvantaged students. However, the criticism is that the resources do not always reach the schools that need them the most (OECD, 2017), not the least schools in economically disadvantaged neighborhoods. Despite differences in economic resources, the neighborhood living environments typically include access to green areas with public parks and gardens, many playgrounds, bicycle paths and sidewalks as means to reduce the car traffic and create better health opportunities for residents.

### 1.5. Study contributions and aims

The local contextual resources, including physical characteristics in contemporary Swedish neighborhoods are not extensively studied specifically in direct relation to child and youth development. Previous research has shown that neighborhood contexts can have both direct and indirect importance to individual development of children and youth across domains of development in several countries (Leventhal and Brooks-Gunn, 2011; Singh and Ghandour, 2012; Odgers et al., 2015; Vinopal and Morrissey, 2020; Gard et al., 2021). Considering the differences in social and cultural context of Swedish neighborhoods compared to those studied in other countries in which SSO methods have been more extensively used (i.e., the United States and United Kingdom), it is important to investigate if neighborhood characteristics can be described in similar ways (if already identified constructs are evident in a Swedish context). Specifically, the aim of the present study was to investigate if aspects of neighborhood conditions, like physical disorder, physical decay and neighborhood dangerousness, could be distinguished and reliably assessed with virtual SSO in a Swedish context. If so, we also wanted to examine if specific neighborhood conditions such as indicators of socioeconomic status on a neighborhood level were associated with the identified neighborhood features. Thus, to the best of our knowledge, the present study is the first study to test the feasibility and psychometric properties of virtual SSO with GSV in Sweden.

Given this theoretical and research background, the overall aim of the present studies was to determine if virtual SSO was a reliable and valid method that can provide assessment of meaningful characteristics of neighborhood contextual conditions that are relevant and reflective of life in the Swedish neighborhoods that were sampled in this study. In two studies, this assessment was performed in neighborhoods within three municipalities in a large (population wise) Swedish city, we: (1) culturally adapted existing SSO related protocols, estimated the inter-rater reliability and construct validity for use in the study context at an item level, and developed virtual SSO measures, as well as, (2) evaluated the criterion validity of the virtual SSO measures by comparison with all residents’ household income on a postal code level.

The aim of Study 1 was to establish whether in-person data collection and virtual data collection using GSV were comparable in a sample of neighborhoods, and if key neighborhood features assessed in-person and virtually could be reliably rated across observers. Thus, the first study intended to review existing SSO related protocols, select and culturally adapt relevant items, develop virtual SSO measures for data collection using GSV, and estimate inter-rater reliability and construct validity in 24 study sites within four neighborhoods.

Research question 1: To what extent is GSV a reliable and valid data collection method, as compared to in-person SSO data collection in the sampled neighborhoods?

Based on the results of Study 1, Study 2 aimed to evaluate the criterion validity of the virtual SSO measures. This was accomplished by, on postal code level, investigating the association between virtually assessed key neighborhood features and socioeconomic status, as indexed by residents’ level of household income.

Research question 2: To what extent are virtually assessed key neighborhood features linked to socioeconomic status (registry data), as indexed by residents’ household income, in the sampled neighborhoods?

## 2. Materials and methods common for study 1 and study 2

In this section, we describe the materials and methods that were common to both studies, after which we describe the study-specific methods and results for study 1, followed by study 2. Further information about the procedure can be found in the Supplementary material Appendices.

### 2.1. Setting

Data were collected in a large Swedish city, in conjunction with a research project which aimed to test the effects of a preschool implemented social emotional learning intervention in a Swedish context (Eninger et al., 2021). The setting included in the present study encompassed three municipalities within the region the city is located, and preschools that were included in the larger research project were situated within these three municipalities. These three municipalities vary, within and between municipalities, in terms of land use, demography and neighborhood characteristics. Due to this variation, the preschool neighborhoods sampled in this study were well suited to examine the study research questions.

### 2.2. Procedure

#### 2.2.1. Identification of study sites within neighborhoods

Across the two studies, neighborhood was defined as the postal code area where the preschools were located (*n* = 22). In Sweden, one of several important administrative boundaries relevant to defining a neighborhood is the community contained within a postal code, and at this level registry data are available to indicate all adult residents’ income as well as educational attainment. Thus, postal code was a key defining feature of the conceptualization of what a neighborhood was in the present study. Another important administrative boundary that has an impact on the local resources and operation of social institutions vital to families and children is at the municipality level. Presently, in Sweden, municipal governments have a wide scale impact on the quality of education and other essential services for children, youth, and families. Thus, the conceptualization of neighborhoods in the present study was based on a consideration of postal codes embedded in particular municipalities within a large Swedish city context.

Geographical distribution and positioning of the postal code areas was obtained from Postnummerservice,^2^ a Swedish provider of geo-demographical data. With support from this web site, we also determined that no modifications or revisions had been made in numbering or geographical distribution for the postal code areas included in the present studies during the seven-year period investigated. Postal code areas were then imported into Google Earth as polygons (shape file format). The size of postal code areas included in the two studies ranged from 0.16 to 7.58 square kilometers, thus, we focused on several selected study sites within each postal code area.

The criteria for selecting sites are further described in Supplementary material Appendix 2. In brief, geographical location and number of sites for a postal code area were selected using the aerial view in Google Earth (GE), scanning for, e.g., land use, type of buildings (business, residential, service), the height of buildings, character of housing areas, and infra structure. Sites were selected out from the criteria that there should be a minimum of four sites per postal code area, and that the combination of sites should represent the variation of qualities within the entire postal code area. For example, if built-up areas in a postal code area consisted of high-rise buildings, detached houses and commercial buildings, the sites were positioned in a manner that this variation in character of housing was captured across sites. The selection of sites was carried out by one person, the first author, who has experience in digital geographical tools as well as in classification of land use from satellite images, and the same procedure was used for all postal code areas and study sites.

**Figure 2:**
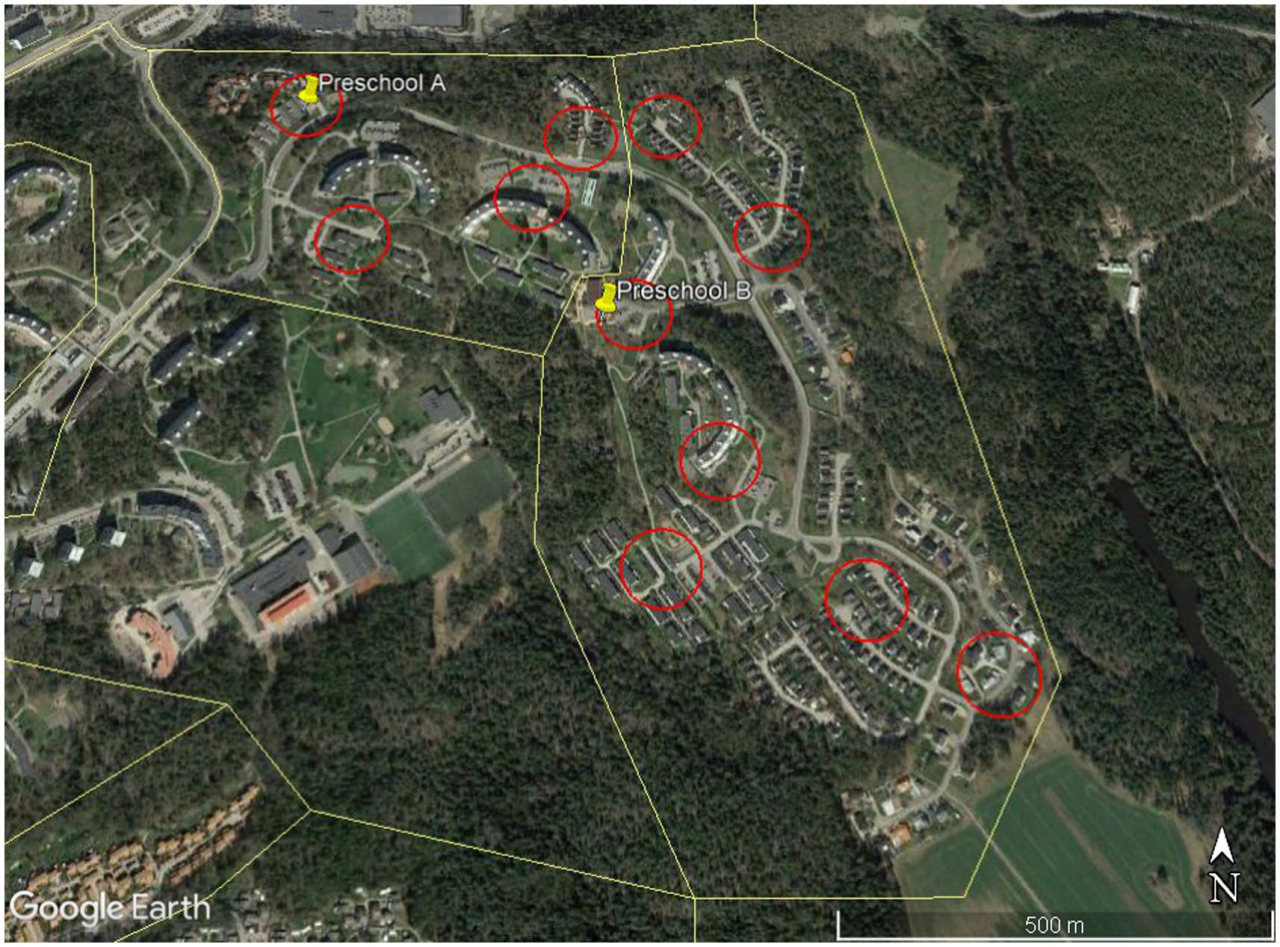
Postal Code Areas (yellow lines) and Study Sites (red circles). Map data: Google Earth, ^©^2020 Landsat/Copernicus.

Based on experiences from previous studies performed in other cultural contexts (Brownson et al., 2004; Hoehner et al., 2005; Clarke et al., 2010; Odgers et al., 2012), the size of each site was set to 50-meter radius (range 50,01–50,99 m). As the aim of the study is to explore if GSV may be a psychometrically sound method for data collection in the sampled neighborhood contexts, we did not choose sites out from GSV availability, image quality, or on what could be detected at street level. However, an initial brief visual inspection was made in GE for a possible variation in GSV coverage in the sampled postal code areas. If possible, GSV coverage was preferred for at least one street within the 100 m circle (checked by using the tool Peg man in Google Earth, GE). Circles indicating each site were drawn with tools in GE, starting with the preschool, whenever possible centered in the first circle. The number of study areas for a postal code area ranged from four to eight sites, varying with the size of the postal code area, see Figure 2 for an example.

In this study, GSV images had a time range of a maximum of 7 years (from the most recent to the oldest image used). For this image time interval, we used the tool for historical satellite images in Google Earth to ensure that no major changes in, for example, infrastructure or housing had occurred within the relevant postal code areas during the study image time span. In Sweden, streets are sometimes kept outside areas where walking areas are defined and separated from motor traffic, as for areas including preschools, schools and residential buildings. Hence, preschools and relevant neighborhood features may be only partially visible as the GSV images are taken with a car-mounted camera. As there seems to be variation in the GSV coverage (also within the postal code areas), we considered it necessary to include an item in which raters estimated the GSV coverage for each study site (responses: Less than 25%; 25–49%; 50–75%; More than 75%).

#### 2.2.2. Development of culturally adapted virtual SSO protocol

The process of developing a culturally adapted virtual SSO protocol was undertaken as follows: first, existing SSO related protocols (e.g., Sampson and Raudenbush, 1999; Odgers et al., 2009; Quinn et al., 2016) were reviewed and items deemed suitable for a Swedish context were identified and selected by the research team; second, identified items were culturally adapted and piloted; third, based on results of the pilot test, a preliminary version of a virtual SSO protocol to be used in a Swedish context was assembled.

##### 2.2.2.1. Review of scale

The process for item development across studies is described in Supplementary material Appendix 1, Figure 1 and the figure overview. This process is briefly summarized here. Several items used in the present study were used verbatim (with translation from English to Swedish) or were adapted from the SSO Inventory: Tally of Observations in Urban Regions (Odgers et al., 2009). The Odgers et al. (2009) SSO Inventory is a comprehensive and thorough instrument that has been described in detail previously (Odgers et al., 2009, 2012). The SSO Inventory has been tested for reliability and validity in the United Kingdom (Odgers et al., 2012), and for reliability the United States(Kepper et al., 2017). For the Virtual SSO Measures Physical Disorder, Physical Decay and Neighborhood Dangerousness, Odgers et al. (2012) reported a moderate to strong level of agreement between raters (observed agreement over 60%, kappa 0.19–0.55, and intra-class correlations (ICC) range 0.72 to 0.85), and positive and significant correlations between all virtual SSO scales and neighborhood SES in 120 neighborhoods in the South and Mid UK. In a study performed by Kepper et al. (2017) in urban and rural neighborhoods in Southern part of the United States, kappa coefficients ranged from 0.04 to 1.00 for items in the domains Physical Disorder, Physical Decay and Safety. For the same items, percentage of agreement between raters was comparable between direct observation and virtual observation with GSV at street-level in 42 street segments, and ranged from 53 to 98 percent (Kepper et al., 2017). For the present study, the first step in the item development process (see Supplementary material Appendix 1 for details) resulted in a total of 78 items intended to assess, for example, physical disorder, physical decay, neighborhood dangerousness, recreational facilities, types of buildings, types of business and service, street safety, and signs. Items that, at face value, were considered to be of little relevance or rarely observed in Swedish neighborhoods were omitted, such as: conditions of streets and sidewalks; culturally specific amenities and business, occurrence of burned out, boarded up or abandoned buildings. In Sweden, conditions of streets and sidewalks are generally good and abandoned, and burned out or boarded up buildings are very rare.

##### 2.2.2.2. Pilot study

The preliminary virtual SSO protocol of 78 items was administered in a field survey and pilot study that served to investigate the items included based on their discrimination in a Swedish context, and to identify observable, physical features relevant for child and youth development in Swedish neighborhoods. First, a field survey was conducted, where the first author walked through the immediate area (approximately 400 m × 400 m) surrounding a preschool, to confirm that items in the preliminary coding protocol could be identified and observed in-person on a street level. Second, a pilot study was performed by two raters with good knowledge about the district; the first author and one psychology student engaged in the larger research project, both female adults and native-born in the city included in the present studies. The raters first carried out an inter-rater calibration, both in-person and with GSV, and then independently performed data collection on the same sites, for a total of 12 sites within a 200 m radius circle around one preschool in each of the three municipalities. The preschool neighborhoods in which the pilot study was carried out were not included in Study 1 and Study 2 but located in the same three municipalities. Geographical location for the sites was selected as described in the previous section.

Item-level inter-rater reliability was assessed using Fleiss kappa for dichotomous items and interclass correlation coefficients (ICCs) for items with multiple-choice responses. Construct validity across methods was assessed using Pearson’s correlation. Items were excluded if inter-rater reliability was considered moderate or less, i.e., kappa below 0.60 (Landis and Koch, 1977). Items were also excluded if construct validity across methods was non-significant, or if items could not be observed, had no variation, or were deemed as not relevant in a Swedish context by the two raters (in total 30 items). Items that were considered non-applicable for Swedish context concerned, for example, if commercial or industrial units were badly deteriorated, ratings of the quality of cars visible on the streets, and if street signs appeared to be almost unreadable due to being badly faded or vandalized.

At this step the Virtual SSO protocol included 48 items and covered items in domains including: physical disorder (4 items); physical decay (6 items); neighborhood dangerousness (2 items); residential units (6 items); business and service (12 items); recreation (3 items); street layout/safety (8 items); signs (3 items); communication (2 items); and rater’s perception of overall condition (2 items). In addition, there were items for imagery, metadata, and two open-ended questions where raters could make notes about (1) the study site’s suitability for families and children, and (2) specific conditions or general observations for the neighborhood. Cultural adapted items that were included were, for example, recycling sites, and if snow or dense vegetation affected the coding in GSV (yes/no).

#### 2.2.3. Observation training and calibration

After determining the items to be used for studies 1 and 2, two raters (not involved in pilot study data collection) attended a one-day training lead by the first author, and held *via* an on-line meeting platform. The raters were two psychology students (one man, one woman, young adults) who were living in other municipalities but in neighborhoods similar to those where data collection was carried out. i.e. the raters had good knowledge of the Swedish neighborhood context. At the training, raters were introduced to the Virtual SSO protocol, the process for data collection, and the tools available in GE and GSV. Inter-rater calibration was performed in GSV at two pre-selected sites, with different neighborhood qualities and located in different municipalities, where raters thoroughly coded different street segments together in GSV until scores across raters converged. The session was recorded and the recordings were accessible to the training participants.

### 2.3. Data collection

All observation and coding were conducted within each 100 m diameter site. Features of significance for children and youths’ movements (e.g., sports fields, play grounds, schools and preschools) that were visible just outside the site boundary, were noted but not coded. An inter-rater reliability check was made at completion of 18% (*n* = 24) of the total number of study sites for Study 1 and Study 2 (*n* = 133). For these initial sites, the two trained raters made separate, independent observations and coding at the same sites. Data collection was performed consecutively for Study 1 and Study 2, between June and October 2020.

## 3. Study 1 materials and methods

Study 1 aimed to establish if GSV was a reliable and valid data collection method, and comparable with in-person data collection for SSO in the sampled neighborhoods. Thus, this first study included in-person and virtual data collection, an item-level analysis of inter-rater reliability and construct validity across the two data collection methods, and development of virtual SSO measures.

### 3.1. Study sites

Study sites in the first study were sites within a sub-sample of the total number of postal code areas (*n* = 22), which were selected in two steps. First, we selected postal code areas suitable for cross-method comparison based on the following criteria: (1) no considerable changes in land use, build-up areas or infra structure between the date of GSV images and data collection, (2) all items included in the virtual SSO protocol, should be able to observe, both with in-person and virtual data collection method. Out of the total 22 postal code areas, 17 postal code areas fulfilled the criteria. Second, from these 17 areas, four postal code areas were semi-randomized for cross-method comparison: three areas (one in each municipality) were randomized; one was added based on physical distance from the others. The four postal code areas included a total of 24 sites, each 50 m radius in size.

### 3.2. Procedure

Study 1 was performed in three steps. First, the two trained raters performed data collection using the final, culturally adapted Virtual SSO protocol to assess physical neighborhood conditions at 24 sites within four postal code areas. Both raters did separate, independent in-person and virtual observations, resulting in a total of four ratings (two in person and two virtual) for all 24 sites. In-person data collection was performed at the same day for all postal code areas, to minimize the risk that street level conditions at a site would differ between raters. To minimize rater bias and to be able to determine the reliability both between raters and across methods for the same areas, conditions were counter balanced across method, rater and neighborhood. Rater 1 performed virtual data collection for two of the four postal code areas before in-person data collection, and virtual data collection for the remaining two postal code areas after in-person data collection. Rater 2 used the same procedure, but postal code areas for virtual data collection before and after in-person data collection were switched compared to rater 1. Second, on an item-level, we estimated inter-rater reliability and construct validity across in-person and virtual neighborhood classification. Number of sites included in the analysis varied due to absence of buildings, or that item was coded by raters as “None visible/cannot evaluate.” Third, we developed scales for Physical Decay, Neighborhood Dangerousness and Physical Disorder for in-person and GSV data, and calculated construct validity across methods. In order to develop a parsimonious scale, composed of virtual SSO measures that are comparable with previous research in other cultural contexts (i.e., Odgers et al., 2012), we proceeded the present study with a focus on seven items that correspond with items within the Virtual SSO measures reported by Odgers et al. (2012).

### 3.3. Measures

In Study 1 we focused on items that correspond with items used within Virtual SSO measures reported by Odgers et al. (2012). Accordingly, based on seven items, we developed three virtual SSO scales described below.

#### 3.3.1. Physical decay

Two items for Physical decay scale were coded; the general condition of the buildings at the location (rated on a three-point scale: 1 = well-kept, 2 = moderately well-kept, 3 = poorly kept), and the condition of the majority of residential units at the location (rated on a three-point scale from: 1 = good condition, 2 = fair condition, and 3 = poor condition). A physical decay scale score was created as mean score for in-person data (Cronbach’s alpha 0.961), and GSV data (Cronbach’s alpha 0.948), with higher scores indicating more physical decay.

#### 3.3.2. Neighborhood dangerousness

Neighborhood dangerousness was assessed with two items covering raters’ perception of whether: the neighborhood was “a safe place to live”; if they “would feel safe walking in this neighborhood at night” (both rated on a five-point scale: 1 = definitely safe, 2 = fairly safe, 3 = unsure, 4 = fairly unsafe, 5 = definitely unsafe). A mean score for these two items was calculated for in-person data (Cronbach’s alpha 0.995) and for GSV data (Cronbach’s alpha 0.981), with higher scores indicating more perceived neighborhood danger.

#### 3.3.3. Physical disorder

Three items intended to assess physical disorder were measured as the presence of: graffiti or graffiti that has been painted over (coded 0–1); abandoned and/or run-down cars or cars with broken windows (coded 0–1); strewn garbage, litter or broken glass on streets or public places (rated 1–4: none, light, moderate, heavy; dichotomized for analysis). Based on these three items, a sum score for physical disorder was calculated for in-person data and for GSV data in which higher scores indicate more physical disorder.

### 3.4. Statistical analysis

All statistical analyses were performed using SPSS Statistics (version 27 for Windows, 2020, IBM Inc., Chicago, IL). On an item level, inter-rater reliability was analyzed using Fleiss Kappa, Intra Class Correlation (ICC) and percentage of agreement across raters. Requirements for inter-rater reliability were values at or above 0.60, analyzed with Fleiss kappa (Landis and Koch, 1977) for dichotomous items and ICC (Shrout and Fleiss, 1979) for items with multiple-choice responses. Kappa values were interpreted as presented in Landis and Koch (1977): 0.41–0.60 (moderate agreement); 0.61–0.80 (substantial agreement); 0.81–0.99 (almost perfect agreement). However, although kappa values may be low (e.g., <0.40) due to low prevalence, corresponding inter-rater agreement can be high, e.g., above 80% (Feinstein and Cicchetti, 1990). Therefore, items with kappa or ICC values near or just below 0.60, were analyzed for percentage of agreement between raters (number of sites agreed/total number of sites), with a cut off at 70%.

Mean values of rater’s scoring was calculated separately for in-person and GSV data, and construct validity across methods was estimated with Pearson’s correlation. Items were retained if requirements were met, i.e., items had kappa/ICC >0.60 or percentage of agreement >70%, for virtual as well as in-person data collection, and a significant correlation (*r*) across methods. In addition, on an item level, we analyzed inter-rater reliability across raters and methods using kappa and ICC with the same cut-offs as described above, specifically: between in-person ratings by rater 1 and GSV ratings by rater 2; between in-person ratings by rater 2 and GSV ratings by rater 1. In total 37 items of the 48 items included in the data collection met the criteria and were retained. Of the 37 items, seven items were used for further analysis and for development of virtual SSO scales (for more details about reduction of items, see Supplementary material Appendix 1, Figure 1). Where appropriate, items were reversed, and sum score and mean scores were calculated to compile SSO measures for in-person and GSV data. Internal consistency and construct validity across methods for the scales were estimated.

## 4. Study 1 results

Results of inter-rater reliability (across raters and across raters and methods), internal consistency, and construct validity (within raters, across methods) are presented in Table 1.

**Table 1:** Inter-rater reliability, internal consistency, and construct validity, for in-person and virtual data.

		In-person					Virtual (GSV)					Cross method		
				Inter-rater reliability		Internal consistency			Inter-rater reliability		Internal consistency	Construct validity	Inter-rater reliability	
													R1In-person – R2GSV	R2In-person – R1GSV
**Scale**		Mean (SD)^a^	N (sites)	ICC^b^/Kappa^c^	Observed agreement^d^	Cronbach’s alpha	Mean (SD)^a^	N (sites)	ICC^b^/Kappa^c^	Observed agreement^d^	Cronbach’s alpha	Pearson correlation (r)^e^		
Item														
	**Physical decay**					**0.961**					**0.948**	0.829***		
1	Condition of buildings	1.826 (0.748)	23	0.791***			1.750 (0.632)	22	0.796**			0.865***	0.767**	0.871**
2	Condition of residential units	1.925 (0.613)	20	0.788***			1.833 (0.594)	18	0.880***			0.705***	0.777**	0.676*
	**Neighborhood dangerousness**					**0.995**					**0.981**	0.915***		
3	Unsafe to live?	2.125 (1.125)	24	0.918***			2.458 (0.977)	24	0.717***			0.934***	0.707**	0.910**
4	Unsafe to walk at night?	2.188 (1.159)	24	0.914***			2.479 (1.058)	24	0.812***			0.872**	0.744**	0.873**
	**Physical disorder**					**n.a.**					**n.a.**	0.464*		
5	Graffiti/graffiti painted over	0.750 (0.361)	24	0.385	75.0%		0.438 (0.425)	24	0.408	70.8%		0.531**	0.185	0.510*
6	Garbage, Litter on streets/public spaces	0.750 (0.442)	24	0.772***			0.708 (0.464)	24	0.789***			0.695***	0.740**	0.792**
7	Abandoned cars	n.a.	24	n.a.	95.8%		0.021 (0.102)	24	n.a.	100%		n.a.	n.a.	n.a.

Virtual SSO Measures in bold. *p* values * < 0.05, ** < 0.01, *** < 0.001.

a Mean for Rater1 and Rater2.

b ICC (Shrout and Fleiss, 1979) for multiple-choice responses: items 1 to 4; 6.

c Fleiss Kappa (Landis and Koch, 1977) for dichotomous responses: items 5; 7.

d Percentage of agreement on item level.

e Between in-person and GSV data collection methods.

Inter-rater reliability for both in-person and virtual data collection methods were found to be between 0.788 to 0.880 for items in the Physical Decay domain (items 1 and 2). Inter-rater reliability for items in the Neighborhood Danger domain (items 3 and 4) were above 0.914 for the in-person method and between 0.717 and 0.812 for data collection with GSV. For items in the Physical Disorder domain, inter-rater reliability was substantial for garbage or litter on streets and public places (item 6). Presence of graffiti or graffiti painted over (item 5) had kappa below 0.41 but above 70% agreement for both methods, and was kept for further analysis. Few abandoned cars (item 7) occurred in the 24 sites, and inter-rater reliability could not be calculated. However, agreement between raters was above 95%, and the item was kept for further analysis.

Internal consistency for the two domains (neighborhood danger and physical decay), in which the response options were suitable for a measure of internal consistency, were above Cronbach’s alpha of 0.948.

Regarding construct validity across methods in person versus GSV, both items and scale score for Physical Decay and Neighborhood Danger correlated significantly, with correlation above *r* = 0.829 except for item 2 that had correlation *r* = 0.705. On domain/scale score level, Physical Disorder had a significant, moderate correlation (*r* = 0.464) between methods. On item level, a strong, significant correlation (*r* = 0.695) was found for garbage and litter (item 6), and graffiti/graffiti painted over (item 5) showed a significant, moderate correlation (*r* = 0.531). Construct validity for abandoned cars (item 7) could not be calculated, due to too few occurrences within the 24 sites.

Inter-rater reliability for rater’s estimation of GSV coverage for each site, was significant (*r* = 0.766). Regarding inter-rater reliability across raters and methods (i.e., Rater1 in person vs. Rater2 with GSV, and vice versa) items in the Physical Decay domain and in the Neighborhood Danger domain were above 0.676 for both conditions. For the Physical Disorder domain, the item for presence of graffiti or graffiti painted over had inter-rater reliability of 0.510 for the condition Rater2 in person – Rater1 GSV, while the condition Rater1 in person – Rater2 GSV was non-significant. Inter-rater reliability for the item for garbage or litter on streets and public places was above 0.740 for both conditions.

## 5. Study 2 materials and methods

For the second study, the research question was to establish if virtually assessed key neighborhood features were linked to socioeconomic status in the sampled neighborhoods. Therefore, we rated neighborhoods using the GSV method and the scales developed in Study 1, and evaluated criterion validity of the virtual SSO measures, by comparison with socioeconomic status, as indexed by level of income of all residents as the postal code level, i.e., in the same neighborhoods/postal codes.

### 5.1. Study areas

Study areas were in total 137 sites (including the 24 sites in study 1) in 22 postal code areas. GSV imagery was not available for four sites, resulting in a final sample of 133 sites for Study 2. Raters estimated a GSV coverage of at least 50% for near half of the sites, and for 113 sites the estimated GSV coverage was 25% or more. Thus, image coverage at street level was considered satisfactory for the sample (N = 133).

### 5.2. Procedure

Study 2 included virtual data collection with GSV only, which was carried out by the same two raters as in Study 1. Rater 1 performed data collection at 61 sites and rater 2 collected data at another 76 sites, i.e., a total of 137 sites with an average of 6.23 sites (SD = 1.23) per postal code area. First, internal consistency for virtual SSO measures was estimated. One item, presence of abandoned and/or run-down cars, occurred in only two of 133 sites and so did not contribute to the measure of physical disorder, thus, this item was excluded from further analysis. Contribution to disorder for the item measuring graffiti was also infrequently observed, however, graffiti was kept as a single item for further analysis, based on its importance for measurement of physical disorder in previous studies (Odgers et al., 2012; Quinn et al., 2016; Marco et al., 2017). Second, site-level measures for Physical Decay, Neighborhood dangerousness and Physical Disorder (now measured as two single items, see below), were aggregated on postal code level. Third, aggregated data were linked to level of household income for all residents living in the postal code areas (registry data). Number of sites included in the analysis varied due to absence of buildings, or that item was coded by raters as “None visible/cannot evaluate.”

### 5.3. Measures

Based on previous presented arguments, we adjusted the measures developed in Study 1, resulting in the final virtual SSO measures presented below. These SSO measures were then used for evaluation of the criterion validity in terms of resident level of income at the postal code level.

#### 5.3.1. Physical decay

Two items for Physical decay were coded; the general condition of the buildings at the location (rated 1–3: well-kept, moderately well-kept, poor kept), and the condition of the majority of residential units at the location (rated 1–3: good condition, fair condition, poor condition). A physical decay scale was created as mean score (Cronbach’s alpha 0.855), higher scores indicating more physical decay.

#### 5.3.2. Neighborhood dangerousness

Neighborhood dangerousness was assessed based on raters’ perception of whether the neighborhood was “a safe place to live,” and if they “would feel safe walking in this neighborhood at night” (rated 1–5: definitely safe, fairly safe, unsure, fairly unsafe, definitely unsafe). A mean score for these two items was calculated (Cronbach’s alpha 0.981), where higher scores indicate more perceived neighborhood danger.

#### 5.3.3. Physical disorder

Physical disorder was measured as two single-items: the presence of graffiti or graffiti that has been painted over (coded 0–1); strewn garbage, litter or broken glass on streets or public places (rated 1–4: none, light, moderate, heavy). This scale score is the sum of these two items, with a higher score indicating more physical disorder.

#### 5.3.4. Level of income

Postal code areas were divided into a category of high-or low-income areas. Of the 22 postal code areas included in the current study, 10 postal code areas were in the first wave (year 2014) in the larger research project (Eninger et al., 2021), and 12 postal code areas were in the second wave (year 2016). The partition of postal code areas was based on mean income in the Region in which the large city was located for the years 2014 and 2016, i.e., 533,475 Swedish crowns, and 580,675 Swedish crowns, respectively. Data for household mean income during years 2014 and 2016 for residents living in the postal code areas was obtained from Statistics Sweden. Across the sample, high-income areas (*n* = 13) were estimated as postal code areas where household mean income for all residents was above the mean income for the region, and similarly, low-income areas (*n* = 9) were postal code areas with a resident household mean income below mean income for the region.

### 5.4. Statistical analysis

All statistical analyses were performed in SPSS Statistics (version 27 for Windows, 2020, IBM Inc., Chicago, IL). In Study 2, data analysis was conducted for Virtual SSO measures across sites and at the postal code level. We estimated internal consistency (Cronbach’s alpha) for virtual SSO measures (when appropriate), and then aggregated data for all 133 sites into postal code level (22 postal code areas). On postal code level, we evaluated the criterion validity of the virtual SSO measures by a group comparison (independent sample t-test) with groups defined by level of income (high or low).

## 6. Study 2 results

Results for internal consistency for the virtual SSO measures, and group level comparison at postal code level, are presented in Table 2.

**Table 2:** Virtual SSO measures and group level comparison for level of income, at postal code level.

Virtual SSO measures		Internal consistency	Low income neighborhood (*n* = 9)		High income neighborhood (*n* = 13)		Group level comparison		
Scale	N (sites)	Cronbach’s alpha	Mean^a^	SD	Mean^a^	SD	t	df	*p*
Item									
Physical decay	104	0.855	2.160	0.249	1.609	0.199	5.766	20	0.000
Neighborhood dangerousness	133	0.981	3.299	0.534	1.887	0.501	6.327	20	0.000
Graffiti/graffiti painted over^b^	133	n.a	3.555	0.882	3.154	1.951	0.575	20	0.572
Garbage, Litter on streets/public spaces^b^	129	n.a	2.152	0.591	1.573	0.275	3.104	20	0.006

a Mean for low-and high-income neighborhoods, respectively.

b Single items.

An independent samples t-test revealed significant differences between high-and low-income postal code areas, see Table 2. Mean values of scales for observed Physical Decay, Neighborhood Dangerousness and a single item measuring signs of garbage or litter in the streets, were significantly higher in low-income areas (i.e., greater physical decay and neighborhood dangerousness in low income areas) than in high-income areas. However, there was no significant difference for signs of graffiti or graffiti painted over between low-and high-income areas.

## 7. Discussion

Neighborhood contextual resources can be important to the positive development of children and youth (e.g., Fauth et al., 2007; Leventhal and Brooks-Gunn, 2011; Browning et al., 2013; Riina et al., 2013; McCoy et al., 2015; Gard et al., 2021), but also provide settings that support young people in their attainment of personal goals and thriving, and provide developmental assets critical in terms of connection with the community (Tolan et al., 2016). However, physical characteristics in neighborhoods can be difficult to measure and have often been assessed through in-person observation and coding of contextual conditions (Clarke et al., 2010; Mooney et al., 2014). With support of digital geographical tools, observations can increasingly be performed from a distance and with fewer resources than in-person observations. Inspired by previous studies, e.g., the work done by Odgers et al. (2012), our overall aim was to determine if virtual SSO with support of GSV is a reliable and valid method that can provide assessment of meaningful characteristics of neighborhood contextual conditions that are relevant and reflective of life in Swedish urban and suburban neighborhood contexts. Results from our studies indicate that, in this sample of Swedish neighborhoods, the virtual SSO method with GSV was a reliable and valid measure of several key neighborhood features assessing safety, orderliness, and the condition of buildings. The method also provided information about neighborhood assets, within the developmental assets framework there is a conceptual connection to the construct of safety, however, in this case it was measured at a contextual level by raters rather than the perception of youth *via* surveys.

### 7.1. Google street view-A reliable and valid data collection method for SSO in Swedish neighborhoods

In our first study, we wanted to examine to what extent Google Street View was a reliable and valid data collection method for SSO in Swedish neighborhoods, as compared to with in-person data collection. Our findings indicated that a majority of the key neighborhood features assessed in-person and virtually were reliable indicators across raters, with substantial construct validity across methods. Virtual SSO measures for the domains Physical Decay and Neighborhood Dangerousness proved to have substantial inter-rater reliability and high internal consistency, while the same measurements for the domain Physical Disorder were moderate.

A closer look at the different scales, indicated that for Physical Decay, i.e., measures of the quality of housing and to what extent buildings in general are maintained, inter-rater reliability was substantial (above 0.767 for both items, across raters and across methods). Further, internal consistency for the scale, was high (above 0.90) for both in-person and GSV data collection methods. Looking across methods, construct validity for residential buildings, was slightly weaker than for buildings in general (about 0.70 compared to 0.86), which might be explained by the fact that some neighborhoods in our sample did not include any residential units. In addition, in Swedish neighborhoods, areas with residential buildings is often separated from motor traffic, and as GSV images are registered by a car-mounted camera, buildings or residential units may have been only partially visible or visible from a distance.

Concerning the scale Neighborhood Dangerousness, the agreement between raters and across in-person and virtual assessment was substantial. However, interrater reliability was higher when rated in person (above 0.91) than when assessed with GSV (over 0.70, below 0.82). As this measure is based on rater’s perception of the neighborhood (i.e., whether they would feel safe to live in the neighborhood and to what extent they would feel safe walking at night), it might be that aspects of neighborhoods, like if there is a friendly or hostile atmosphere, can be difficult to perceive when rating images virtually.

The picture is a bit more complex for the scale measuring Physical Disorder. Inter-rater reliability was substantial for the measure for presence of garbage or litter on streets and public places, both for in-person and GSV methods, as well as across raters and methods. On the other hand, presence of graffiti or graffiti painted over had fair inter-rater reliability for assessment with both methods, while percentage of agreement was above 70%. In addition, rater’s assessment of graffiti for in-person and virtual methods differed across conditions, in that inter-rater reliability was non-significant for the condition Rater 1/in-person vs. Rater 2/virtually, but significant for the condition Rater 2/in-person vs. Rater 1/virtually. Furthermore, only a few abandoned cars occurred in the 24 neighborhoods included in our first study and this item was found to be non-valid for SSO in the sampled neighborhood contexts. Abandoned cars are uncommon in Sweden and cars are in general of high quality, partly because all vehicles have to undergo a yearly inspection.

High levels of observed agreement between raters about the presence of disorder, physical decay and assessments of neighborhood safety have also been reported for studies comprising neighborhoods in the South and Mid United Kingdom (Odgers et al., 2012) and urban and rural neighborhoods in the Southern United States (Kepper et al., 2017). In the study performed by Kepper et al. (2017), the percentage of agreement between raters was comparable between direct observation and virtual observation with GSV at street-level in 42 street segments, and ranged from 53 to 98% (Kepper et al., 2017). For street segments in 120 neighborhoods, Odgers et al. (2012) reported observed agreement over 60% for virtual SSO measures of disorder, decay and danger, with substantial inter-rater reliability. Our results also align with results from the study by Odgers et al. (2012), where the Neighborhood Dangerousness scale was reported to have the strongest inter-rater reliability (ICC 0.85), followed by the scales for Physical Decay (ICC 0.74) and Physical Disorder (ICC 0.72), respectively (Odgers et al., 2012). In addition, a similar study was performed in another European country, Spain, in which a GSV based observational scale was compared with physical audits (Marco et al., 2017). In 92 small administrative units in a major city in Spain, results showed moderate interrater reliability for scales for physical disorder (ICC 0.43) and physical decay (ICC 0.55), and positive correlations between virtual (GSV) and on-site assessment for physical disorder (*r* = 0.39) and physical decay (*r* = 0.37; Marco et al., 2017).

Like previous studies (Odgers et al., 2012; Kepper et al., 2017; Marco et al., 2017), our study indicated robust estimates of neighborhood dangerousness and physical decay when virtually assessed with GSV. However, measures for physical disorder seems to be of a more complex nature, maybe due to differences in the type of features that occur and can be observed within neighborhoods in different cultural contexts.

Altogether, our first study shows that Google Street View is a reliable and valid data collection method within the sampled neighborhoods and for observations of Physical Decay, Neighborhood Dangerousness, signs of garbage or litter, and presence of graffiti, although with a dubious outcome for the latter. Based on the analysis performed in Study I, we also have a full instrument (37 items) for virtual SSO in a Swedish context. However, we decided to keep a majority of the instrument as descriptive, but have found the scales to be practically useful and psychometrically sound as well as consistent with prior research by Odgers et al. (2012), and thus appropriate for further examination and development in Swedish urban and suburban neighborhoods.

### 7.2. Virtually assessed neighborhood features linked to level of household income in Swedish neighborhoods

In order to evaluate the criterion validity of the virtual SSO measures, in Study 2, we compared four virtually assessed neighborhood features, specifically the scales for Physical Decay and Neighborhood Dangerousness as well as assessment of signs of garbage and of graffiti, with the economic status in the neighborhood, i.e., the residents’ levels of household income on a postal code level. We found that the mean values for observed Physical Decay, Neighborhood Dangerousness and a single item measuring signs of garbage or litter in the streets, were significantly higher in low-income neighborhoods than in high-income neighborhoods. These results are in line with the study by Odgers et al. (2012), in which neighborhoods classified as the most hard-pressed (based on census-derived socio-economic data) also had the highest levels of virtually rated disorder, decay and dangerousness.

Looking at this larger sample of 133 neighborhoods, where assessment was made with GSV only, there was no significant difference between high-and low-income neighborhoods for the mean value for the item “graffiti/graffiti painted over.” In our study of the sampled neighborhoods, the item measuring graffiti was infrequently observed, which is interesting as graffiti has been reported to be of importance for measurement of physical disorder in previous studies (Odgers et al., 2012; Quinn et al., 2016; Marco et al., 2017).

Instead, signs of garbage or litter in the streets and public places was the only one item measuring Physical Disorder that proved to be a useful index in the sampled neighborhoods. Similarly, the item measuring garbage or litter in the streets had highest prevalence of items observed in the neighborhoods investigated by Odgers et al. (2012), and also had the highest frequency of a set of items indicating disorder when assessed with GSV in an urban context in United States (Mooney et al., 2014; Quinn et al., 2016). This could mean that virtual observations of public littering can be an important indicator of neighborhood disorder also in a Swedish context.

Such findings indicate that physical features in the neighborhoods could be important markers of the economic level in the neighborhood, and thus contextual resources where children grow. Indeed, ecological system theories (e.g., Bronfenbrenner, 1979) suggest that neighborhood context, including the quality of the physical features as well as the sense of safety, plays an important role for socialization and for development of children. In neighborhoods where children can feel safe, children can thrive (Leventhal and Brooks-Gunn, 2000; Vinopal and Morrissey, 2020). Measuring the quality of neighborhood or contextual resources is however not easy. For example, subjective measures of perceived aspects of neighborhood provide important information on individuals’ appraisals of the environments they live in. Such assessment may however be subject to several different types of bias (Mooney et al., 2014; Marco et al., 2017) which in turn could be a limitation when investigating child development and health. Objective measures, on the other hand, could capture important aspects in the neighborhood where children grow that subjective measures do not. In that sense, using virtually assessed neighborhood features may open doors to the assessment of contextual resources, that could be used in research on child development. Virtual assessment can also be used as a complement, when suitable, to a diverse array of measures including those that are subjective (youth perceptions and reports), and provide part of the measurement picture of person-context interactions that are important to understanding the complexity of child and youth development.

### 7.3. Cultural considerations

In our study, we defined and operationalized the neighborhood as a postal code area (i.e., zip-code) which is a common way to distinguish between different neighborhood areas in Sweden. Other studies, however, may define and operationalize neighborhood as a concept using other social or geographical proxies, such as land-use, census tract, or zip-code (Dietz, 2002). This is important in terms of bias in comparison of the results between studies in various cultural contexts. For example, the validation study from Spain used census block groups as proxy for neighborhood (Marco et al., 2017) and the US validation study used census tracts to operationalize neighborhood (Kepper et al., 2017). Such differences, although sometimes referred to as minor (Dietz, 2002), may provide some explanation to somewhat differing results in validation of GSV as a SSO method in various cultural contexts, including Sweden.

The importance of discussing study results in a cultural perspective can further be underscored referring to our finding of no differences between low-and high-income neighborhoods in terms of observations of signs of graffiti or graffiti painted over. This finding is interesting as research from other cultural contexts, such as the United States (Quinn et al., 2016), the United Kingdom (Odgers et al., 2012) and Spain (Marco et al., 2017), suggest that graffiti may be one of the most important markers of neighborhood physical disorder. The results regarding the graffiti as a marker for physical disorder in our study, however, does not corroborate such findings and ideas.

As from abolition of the Swedish zero tolerance toward graffiti in year 2014, the Swedish municipal management practices responsible for planning and management of public places have now adopted a broader, yet dichotomous view of graffiti which is also shown in their management of public places in the municipalities. On the one side, municipal management practices often install legal graffiti walls as means for showing graffiti as a work of art rather than a delinquent act. On the other side, the same services have a policy to remove illegal graffiti as quickly as possible as means as bringing order in the public places (Paulsson, 2016). Thus, as graffiti is not uniformly seen as an indicator of physical disorder in Sweden, it may not be a good marker for contextual resources in Swedish neighborhoods.

On the other hand, our results showed that virtually assessed signs of garbage or litter in the streets or public places was the only item in the physical disorder scale for which high-and low-income neighborhoods could be distinguished. Such a finding lends further support to virtual observation of public littering being a strong indicator of neighborhood disorder in a Swedish context. These somewhat inconsistent results in terms of measurement of physical disorder call for cautiousness related to the cultural and legal aspects of neighborhood assets and characteristics. In that sense, in addition to removing and/or adding culturally informed items to the observation protocol, it would be imperative to also consider the laws and regulations, as well as social codes in the cultural context of interest when culturally adapting, implementing, and using objective measures to capture neighborhood resources.

### 7.4. Strengths and limitations

We studied neighborhoods in municipalities located in the wider region of a major Swedish city, and neighborhoods are distributed geographically and with diversity in character and land use, e.g., areas with high-rise buildings or areas with detached houses. Thus, our neighborhoods can be considered as a mix of urban and suburban built-up areas, which is common in a Swedish context, especially outside city center. Streets and sidewalks are in general well-kept in Swedish neighborhoods and you can easily walk or go by bike within and between neighborhoods. In addition, neighborhoods where people live often include a lot of green areas, parks as well as stretch of woodlands, also in close connection with high-rise buildings. The present study findings are situated in this wider context. More research is needed to determine the generalizability of the present study findings to other Swedish neighborhoods, in other large, mid-sized and smaller cities as well as in rural areas. Furthermore, more research is required in order to investigate the criterion validity of the full instrument for virtual SSO in Sweden, as the study findings are limited to the measures of Physical Decay, Neighborhood Dangerousness and Physical Disorder that were included in the present study.

So far, studies that have examined virtual assessment of neighborhood features have often been performed in an urban environment (Sampson and Raudenbush, 1999; Clarke et al., 2010; Quinn et al., 2016), with assessment of block faces (Less et al., 2015; Quinn et al., 2016) or street segments (Brownson et al., 2004; Odgers et al., 2012; Griew et al., 2013; Kepper et al., 2017). Given that Swedish neighborhoods have a somewhat mixed layout of urban and suburban environment, we choose to define neighborhoods as postal code areas and to select study sites within the postal code areas. However, in contrast to previous studies (Griew et al., 2013; Mooney et al., 2014; Bader et al., 2017), we did not use support from other geographical tools (like GIS or CANVAS). In using Google Earth and Google Street View only, we may have selected study sites that not fully capture all qualities in a postal code area. Also, study sites were selected by one single researcher, which could be considered a limitation. As suggested by Less et al. (2015), we yet focused on small study areas (each study site was 100 m in diameter) and on features that were relatively stable over time.

In addition, although raters took a virtual walk down the streets in GSV, carefully looking at key features within each study site, not all streets had full GSV coverage and some features could only be observed from a distance. It is possible that a study with the same virtual SSO coding protocol, but performed in a geographically larger area with even better GSV coverage and including a city center, could provide a larger variation of buildings and other neighborhood characteristics, which in turn could give more information about key neighborhood features relevant in a Swedish context. Further, although different raters were involved in different sub-studies, we did not have enough raters to have sufficient diversity in socio-demographic factors to be able to speak to how raters themselves and their socio-demographic backgrounds may have been important to study ratings. Because the training in the SSO method, in our case, was tailored to a Swedish context, Swedish language fluency and prior residence (knowledge about the physical and cultural context) in a Swedish urban or suburban context would be recommended in future raters using this instrument in a Swedish context. However, even though calibration and training prior to data collection were performed on-line (due to C19-restrictions), agreement between raters and correspondence across methods were good. To further improve the outcome, a recommendation is to meet in person for training and calibration.

In terms of study limitations, the timing of the GSV images did vary (from oldest to newest image observed and rated) and thus these images are not sensitive to short term temporary changes in neighborhood features, which is a widely recognized general limitation of this type of method. Also, the use of GSV images are subject to what is publicly available and the timing of when images are updated (which can be updated by Google on a non-systematic basis). Further, there was also variation of some images time period and the time period of the income data collection. These timing issues are recognized limitation within this field, and has been encountered by other researchers such as Clarke et al. (2010) in which the timing of the in person SSO and virtual SSO differed (4–5 years difference in the timing of data collections, in real life and virtual), and the correspondence between methods for several key factors showed acceptable levels of agreement (e.g., 0.34 to 0.75 levels of agreement for garbage or litter in streets, conditions of residences, and presence of graffiti). In a Swedish context, municipal governments are in charge of the condition of local neighborhoods and the operation of these governments is relatively stable (local governments do change but over the course of several year time intervals). Although GSV is not a precise momentary examination of neighborhood features (e.g., Clarke et al., 2010), long standing features of neighborhoods are likely to be reflected with some stability over the course of several years, and this was the focus of the present study.

Other limitations within the wider field are that measures and scales are not always defined in the same way across cultures, applications and in different disciplines. This makes it difficult to compare reported outcomes, as scales can include similar but often not the exact same items. For example, in our studies we had two items for Physical Decay while Odgers et al. (2012) had five items, and the study by Kepper et al. (2017) included six items in their scale for Physical Decay. Similarly, our scale for Physical Disorder initially consisted of three items, compared to five items (Odgers et al., 2012) and nine indicators (Sampson and Raudenbush, 1999; Mooney et al., 2014; Quinn et al., 2016) reported in other studies. For the scale Neighborhood Dangerousness, the internal consistency was very high, which could indicate that the two items (if raters consider the neighborhood as safe to live in and safe to walk at night) to a large extent overlap. However, other studies have used scales based on the same two items (Odgers et al., 2012; Kepper et al., 2017), with observed agreement between raters exceeding 60% for in-person and virtual assessments.

Although results from both our studies indicate that virtual SSO with GSV is a reliable and valid measure of several key objective neighborhood contextual conditions, this sample of neighborhoods may not have been representative of Swedish neighborhoods in general. A sample where, for example, more hard-pressed neighborhoods, neighborhoods located in city center’s or in rural areas could be included may provide a broader understanding of a Swedish neighborhood context. Further studies with another sample of neighborhoods using the full instrument could also contribute to a broader understanding of the criterion validity for the virtual SSO instrument and may give further information on how the additional items (beyond the seven items included in our analysis) relate to level of income in Swedish neighborhoods.

Our studies also include a limited number of postal code areas for each municipality, based on the location of preschools that were included in the larger research project, and it might be that level of household income for these neighborhoods do not reflect the level of income for Swedish neighborhoods in general. On the other hand, measures for neighborhood socioeconomic status are often income and education at the level of the individual, e.g., from resident survey or parent report, sometimes aggregated to neighborhood level. In contrast, the proxy for socioeconomic status in our study are based on register data for levels of income on neighborhood level, which would give a more direct measure of neighborhood socioeconomic status.

These studies are unique in that they were conducted in a Swedish context, more specifically in municipalities located near a major city. There is a need for further examination of whether the method can be generalized for use in other parts of the world and in different cultural contexts. We believe that our studies make a valuable contribution this wider effort, and to our knowledge, these are the first studies where virtual SSO with GSV has been performed in Sweden.

### 7.5. Implications for future research

The findings of these studies have implications for future research in several ways. Specifically, the development of a culturally adapted, reliable and valid observational instrument sets the stage for more research of this nature, and for furthering our understanding of the complex interplay over time between neighborhood attributes, and, e.g., individual and family factors, in shaping development. For example, more insight could be gained by exploring how neighborhood conditions may contribute to youth development *via* other contextual conditions such as residents’ views of social cohesion, collective efficacy, exposure to crime and violence, as well as other important contexts for youth such as school climate and quality. This could inform the area of positive youth development in terms of broadening the notion of contextual factors to include also neighborhood factors as possible moderators in existing developmental models and moderators of intervention effects (individual level interventions). Thus, the effects of positive youth interventions could be nuanced by including an objective measure of neighborhood conditions.

Keeping in mind that the results of the present studies are correlational, it would be premature, however, to suggest causation in any direction. An investigation of the possible mechanisms involved could be further examined in future studies. For example, in a study performed in another European country (Spain), a spatial analysis indicated that physical decay and physical disorder tend to cluster geographically rather than being randomly scattered (Marco et al., 2017). Previous research suggests that the presence of neighborhood disorder encourages more disorder, and the authors wrote “Even if we wish it were not so, disorder triggers attributions and predictions in the minds of insiders and outsiders alike.” (Sampson and Raudenbush, 1999, p: 604). Consequently, keeping neighborhoods clean can have a positive effect on the behavior of both residents and others. To further analyze the spatial distribution of areas proved to have high levels of physical decay and disorder, could be a way to identify neighborhoods where the physical environment can be improved, by means of cleaning the streets more often and to a larger extent maintain buildings, but also to identify neighborhoods where interventions that promote youth development could be extra fruitful.

It would also be important to combine virtual SSO with further measures of SES, like education level, occupational status, and economic assistance, among residents in a neighborhood to see if these measures also can be spatially associated with neighborhood features. In a study by Marco et al. (2017), performed in a large Spanish city, neighborhood physical disorder and physical decay correlated with lower levels of education and property value at census block group level. This would provide stronger support for the relation between SES and physical features in a neighborhood.

## 8. Conclusion

In a Swedish context, Virtual SSO with GSV was found to be a valid method that offers a possibility to reliably assess key neighborhood physical conditions like condition of buildings, safety and orderliness, in this sample of neighborhoods. Moreover, the method also provided information about contextual resources described in terms of socioeconomic status, as observed key neighborhood features could be linked to residents’ levels of household income.

This study fills a significant gap in the positive development literature by developing observational tools that can measure contextual conditions. The possibilities of this method are significant in that this would allow for the measurement and development of new knowledge about the person context interactions, also in a Swedish context, that are central to many theories of positive development among youth. This, in turn, could contribute to a deeper understanding of how physical neighborhood characteristics affect children’s and youth’s living conditions and potential for development.

## Data availability statement

The datasets presented in this article are not readily available because this study’s ethical review does not allow for study data to be in a public repository. For meta-analysis or confirmation of published study results, deidentified data requests will be reviewed, requests should be made by qualified researchers (e.g., Ph.D.) along with ethical permission under Swedish law regarding secondary data analysis. For a copy of SSO items used the studies in this article, in Swedish, please contact the corresponding author of this publication. Requests should be directed to ICG, ingela.clausen.gull@psychology.su.se.

## Ethics statement

This study was reviewed and approved by the Stockholm Regional Ethics Review Board (dnr. 2018/2334–31/5).

## Author contributions

ICG, LE, and LF-W: methodological design, development of culturally adapted virtual SSO protocol, and data analysis. ICG, SK, ÅN, LE, and LF-W: manuscript conceptualization and writing – original draft. ICG, SK, ÅN, LF-W, TO, and LE: writing, review, and editing. All authors contributed to the article and approved the submitted version.

## Funding

This study was supported by funding from the Department of Psychology, Stockholm university and Centrum för kompetensutveckling inom vård och omsorg (CKVO) [Centre for Competence in Treatment and Care].

## Conflict of interest

The authors declare that the research was conducted in the absence of any commercial or financial relationships that could be construed as a potential conflict of interest.

## Publisher’s note

All claims expressed in this article are solely those of the authors and do not necessarily represent those of their affiliated organizations, or those of the publisher, the editors and the reviewers. Any product that may be evaluated in this article, or claim that may be made by its manufacturer, is not guaranteed or endorsed by the publisher.

## Footnotes

^1^ https://www.google.com/streetview/

^2^ https://postnummerservice.se
