# Supplementary material for: Neighborhood conditions in a Swedish context-Two studies of reliability and validity of virtual systematic social observation using Google Street View
Source: Front Psychol. 2023 Jan 27;14:1020742. doi: 10.3389/fpsyg.2023.1020742 (PMC9911895; doi:10.3389/fpsyg.2023.1020742)
Supplement: Supplementary file 1 [file Data_Sheet_1.docx]

# **Supplementary Material Appendices**

## **Supplementary Material Appendix 1 (SM_A1)**


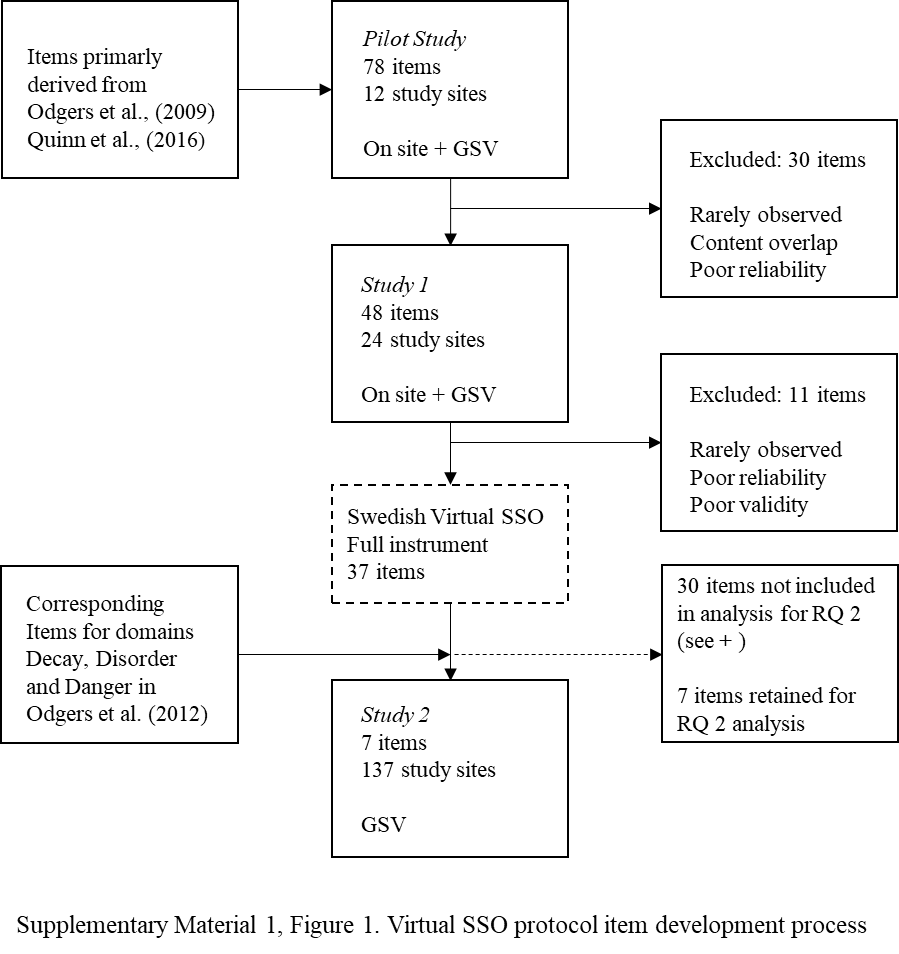


## **Figure A1 Overview**

For the pilot study, we derived 71 items from the SSO Inventory: Tally of Observations in Urban Regions (Odgers et al., 2012), and from a virtual audit of physical disorder (Quinn et al., 2016). Of these 71 items, 48 items were used verbatim, except for a translation of the items from English into Swedish, 23 items were adapted to a Swedish context. An example of item adaptation was to focus on a general rather than a specific feature of a home. For instance, the original item focused on the observer rating features of a front garden of a home. A front garden is uncommon in Swedish homes, and this item was adapted to focus on any visible garden connected to a home, not just front gardens. Another example of an adaptation to a Swedish context was that an item (Quinn et al., 2016) asked the observer to focus on vacant or undeveloped land. In Sweden, within or close to large cities, vacant land is uncommon, and if land is undeveloped, it is often open spaces or forests that are available for all (typically state-owned or owned by a municipality). Thus, this item was culturally adapted to measure presence of areas with naturally growing vegetation. For the pilot study, the remaining seven items were novel, and further items were related to collecting meta-data.

The decision-making guidelines for not retaining items into the next study (entitled Study 1) from the pilot study were the following: 1) items were not observed or rarely observed in the Swedish postal codes examined in the pilot study, 2) if two item domains (i.e., an item domain was viewed as a number of items designed to measure a particular construct) had substantial content overlap, we retained one domain rather than keeping multiple domains that were content redundant with one another, and 3) if items showed poor inter-rater reliability for either on-site or GSV ratings (poor inter-rater reliability cut offs are described in the article), they were not brought forward into the next sub-study (i.e., study 1).

For study 1, there were 48 items. Based on the results of study 1, 11 items were not brought forward into study 2, and reasons for not bringing an item forward included: 1) items were not observed or rarely observed in the Swedish postal codes examined and/or 2) items showed poor inter-rater reliability for either on-site or GSV ratings, and poor construct validity across methods. Thus, the finalized Virtual SSO protocol consisted of 37 items (retained from the pilot study and study 1 results).

**+ =** Of these 37 items, nine items concerned physical disorder (3 items), physical decay (4 items), and neighborhood dangerousness (2 items). Scales were created based on these nine items, of which in the physical decay scale two items were not included in the scale due to a high number of missing observations. This then left a total seven items that corresponded to items in Odgers et al. (2012). Further, these seven items also showed good inter-rater reliability and good internal consistency for the scales, and were retained for the analysis to test Research Question 2. In the finalized virtual SSO instrument, in addition to the seven items described (physical disorder, physical decay, and neighborhood dangerousness) there were also 30 items which are of a descriptive nature and were not included in the analyses for Research Question 2. These 30 descriptive items concern the presence of types of buildings, street features, signs, areas of recreation, business and services in the area, quality and coverage of GSV imagery, metadata (e.g., time stamp of image), and open-ended area for observer notes, as well as raters’ perception of overall conditions in the areas observed.

In the data availability statement of the article, the SSO items for the pilot study, study 1, and study 2 are available by request to the corresponding author.

# **Supplementary Material Appendix 2 (SM_A2)**

## **Criteria for choosing study sites**

1. Study sites were chosen using a combination of the aerial view in Google Earth (GE), and image availability in Google Street View (GSV). The aim was that the combination of all study sites in a given postal code area would be representative of the variation of qualities and land use within an entire postal code area.
2. In GE, we obtained an overview of the land use within the postal code area by scanning for example residential areas, industrial or business areas, railways or motorways, general height of buildings and larger areas of green space, forests or bodies of water.
3. We performed an initial visual inspection of GSV coverage within the postal code area, using the tool “Peg man” in GE. By pressing Peg Man, blue lines were highlighted in the aerial view, showing streets where GSV images were available. GSV coverage was preferred for at least one street within each study site.
4. We choose position for each study area in a way that different types of relevant features, like high-rise buildings, low-rise buildings, commercial buildings, type of residential buildings, infra structure and land use, within the postal code area were represented across study sites. In GE, we used the zoom function to get a better view of the features present in the postal code area. If the postal code areas included large areas of forests or green space, these features were preferable included within a circle indicating a study site, rather than to place a whole study site in the forest, as GSV images were not likely to be available where there were no roads. See Figure 2 in the article for an example of position of study sites.
5. The study sites were positioned within the boundaries of the postal code areas, and the study sites in a postal code ranged from four to eight study sites, with at least 50% of the site within the boundaries. Circles indicating each study site were drawn with the ruler tool in GE, and the size of each site was set to 50-meter radius (range 50,01 – 50,99 m) starting with the preschool, whenever possible centered in the first circle.
6. In GE, we saved each circle (as Places, one folder for each postal code area) with a unique name and number, e.g. Preschool A #1 for the first study site in the postal code area where preschool A is located.
7. For each postal code area, we allocated study sites and ensured that all features that were to be classified were included in any of the study sites, and that there was GSV coverage for at least one street within each study site.

# **References Cited in the Supplementary Material Appendices**

Odgers, C. L., Caspi, A., Bates, C. J., Sampson, R. J., & Moffitt, T. E. (2012). Systematic social observation of children’s neighborhoods using Google Street View: A reliable and cost-effective method. *Journal of Child Psychology and Psychiatry and Allied Disciplines*, *53*(10), 1009–1017. https://doi.org/10.1111/j.1469-7610.2012.02565.x

Quinn, J. W., Mooney, S. J., Sheehan, D. M., Teitler, J. O., Neckerman, K. M., Kaufman, T. K., Lovasi, G. S., Bader, M. D. M., & Rundle, A. G. (2016). Neighborhood physical disorder in New York City. *Journal of Maps*, *12*(1), 53–60. https://doi.org/10.1080/17445647.2014.978910
